# Relevance of a skilled vascular surgeon and optimized facility practices in the long-term patency of arteriovenous fistulas: a prospective study

**DOI:** 10.1590/2175-8239-JBN-2018-0125

**Published:** 2019-04-11

**Authors:** Esteban Lucas Siga, Noemi Ibalo, Maria R. Benegas, Farias Laura, Carlos Luna, David H. Aiziczon, Elvio Demicheli

**Affiliations:** 1 Dialisis Madariaga Buenos Aires Argentina Dialisis Madariaga, General Carlos Madariaga, Buenos Aires, Argentina.; 2 Hospital Interzonal de Agudos Buenos Aires Argentina Hospital Interzonal de Agudos, Mar del Plata, Buenos Aires, Argentina.

**Keywords:** Arteriovenous Fistula, Nephrology Nursing, Vascular Patency

## Abstract

**Introduction:**

Arteriovenous fistulas (AVF) are the best hemodialysis vascular accesses, but their failure rate remains high. Few studies have addressed the role of the vascular surgeon's skills and the facility's practices. We aimed to study these factors, with the hypothesis that the surgeon's skills and facility practices would have an important role in primary failure and patency rates at 12 months, respectively.

**Methods:**

This was a single-center, prospective cohort study carried out from March 2005 to March 2017. Only incident patients were included. A single surgeon made all AVFs, either in the forearm (lower) or the elbow (upper). Vascular access definitions were in accordance with the North American Vascular Access Consortium.

**Results:**

We studied 113 AVFs (65% lower) from 106 patients (39% diabetics, 58% started with catheter). Time to first connection was 21.5 days (IR: 14 - 31). Only 14 AVFs (12.4%) underwent primary failure and 18 failed during the first year. Functional primary patency rate was 80.9% (SE 4.1) whereas primary unassisted patency rate, which included PF, was 70.6% (4.4). Logistic regression showed that diabetes (OR = 3.3, 95%CI 1.38 - 7.88, p = .007) and forearm location (OR = 3.03, 95CI% 1.05 - 8.76, p = 0.04) were predictors of AVF failure. Patency of lower and upper AVFs was similar in non-diabetics, while patency in diabetics with lower AVFs was under 50%. (p = 0.003).

**Conclusions:**

Results suggest that a long-lasting, suitable AVF is feasible in almost all patients. The surgeon's skills and facility practices can have an important role in the long term outcome of AVF.

## Introduction

It is well known that arteriovenous fistulas (AVF) have higher patency rates and fewer complications than arteriovenous grafts and central venous catheters^1^. Therefore, all clinical guidelines have favored their use since 1997^2^^-^4.

The major pitfall of AVF is the high incidence of primary failure and failure to mature^5^. There are many factors that contribute to AVF failure: age, female gender, diabetes, and AVF location could be mentioned as very relevant. The role of the case mix characteristics and AVF location have been widely assessed and recently summarized in a systematic review^6^. However, two important factors should also be taken into account: the experience of the vascular surgeon and the so-called "facility practices" such as nursing skills, time to first cannulation, vascular access surveillance by a dedicated team, etc.

The importance of these factors has been acknowledged over the past years^7^. However, there are very few studies that describe the activity of only one surgeon. Furthermore, the vascular surgeon factor is usually studied isolated from the facility practices. We are not aware of any study that assessed both factors jointly and a single surgeon operating with the same staff throughout the duration of the study.

Aiming to study these factors, we propose the following work hypothesis: 1) The surgeon's skills would have a relevant role in the primary failure rate, and 2) The facility practices would have an important role in the functional primary patency rate during the first year of AVF use.

We hereby submit the results obtained by a single vascular surgeon working with the same equipment and team during a 12-year period. The main outcomes were primary failure rate and functional primary patency rate during the period. The secondary outcome was the influence of major risk factors on primary unassisted patency rate.

## Methods

This was a prospective study, performed in accordance to the Declaration of Helsinki. The database only included new consecutive incident patients - from March 2005 to March 2017 - whose first AVFs were made in upper extremities by the same vascular surgeon. When an AVF showed primary failure and a new access was made, the latter was included only if it was in the other upper extremity since we cannot exclude a modification of the vasculature of the first extremity even if the AVF did function for a few hours. Exclusion criteria were: a) younger than 16 years, b) coagulation disorders before starting dialysis, c) vascular access other than an autologous AVF in an upper extremity.

The vascular access location and type were entirely selected by a senior vascular surgeon, with ten years of experience before the start of the study. A radiocephalic fistula (lower) at the wrist was the first option for AVF creation. A brachiocephalic fistula (upper) was chosen if a radiocephalic AVF could not be placed.

The decision was guided only by clinical criteria and a physical examination. The AVF accesses were performed with a "side-by-side technique". In the wrist, the distal end of the vein was ligated creating a functional side-to-end anastomosis. Before the ligation, the distal end was used to introduce a thin catheter (2 mm diameter and 45 cm long) to infuse a mixed solution of heparin (5000 UI) and 500 cc of saline solution in order to expand the vein and artery, and to check the permeability of the vein vessels. In the brachiocephalic fistula, the median cubital vein and the communicating branch between deep and superficial systems were always ligated, whereas the flow of the efferent vein was preserved.

The newly created access was followed-up at one-week intervals by the senior nephrologist. A systematic physical examination was conducted in each consultation. After collecting essential information, a detailed examination of the anastomosis and the body of the fistula was conducted, primarily focused at the thrill and pulse. There was no pre-established criterion for the first cannulation. The date and technique were always chosen by the senior nephrologist, based in a thorough physical examination. Fistula maturation was defined as the time from access creation to the cannulation of the fistula, assessed by the senior nephrologist. Before the first puncture, an experienced staff technician was exclusively assigned to connect the AVF during the first three months. In the first session, the blood pumped at a maximum of 150 mL/min. If the patient had a catheter, the first cannulation was with a 17G needle for venous return only.

The following definitions were used: *Immediate Vascular Access Failure* (primary failure, PF), in which an access had no immediate bruit or thrill or these were lost within 72 hours of creation^7^; *Functional Primary Patency* (FPP), which was the time from the first successful two-needle cannulation until any first intervention (endovascular or surgical) to maintain or restore blood flow, or reaching the end of study period^8^. *Primary Unassisted Patency*, which was the time of access creation or placement until any first intervention (endovascular or surgical) to maintain or restore blood flow, or reaching the end of study period^7^.

*Statistical analysis*: Data are reported as mean and 95% confidence interval (CI), median and interquartile range (IR), or percent and frequency, as appropriate. We analyzed the independent association between AVF failure in the first year after its creation and selected risk factors: diabetes (DBT), female gender, AVF location (upper or lower) and catheter use at the time of the AVF creation by univariate logistic regression analysis. If the unadjusted odds ratio (OR) was significant for a risk factor, it was used as a covariate to analyze the remaining risk factors by multivariate logistic regression. We estimated functional primary patency and primary unassisted patency rates and their standard errors (SE) with the Kaplan Meier analysis and compared them with the log-rank test. We made the calculations with the Microsoft Office Excel(tm) data analysis tool and Epidat 4.2 free software^9^. We defined significance as a two-sided *p* value < 0.05.

## Results

We included 106 out of 132 patients. Except for 4 aboriginals, all of the patients were Caucasian. Table 1 shows the patients' main demographic characteristics. It was a high-risk population, with a high percentage of diabetics, and severe anemia and hypoalbuminemia when dialysis started. In addition, 58% of the sample began hemodialysis with a temporary catheter.

**Table 1:** Demographic characteristics

Number of patients	106
Age; median (Interquartile Range)	60.4 (47.1 - 66.8)
Female gender; number (%)	35 (33)
Etiology; number (%)	
Diabetic	41 (39)
Unknown	25 (23.6)
Autoimmune	14 (12.9)
Vascular	10 (9.4)
Genetic	9 (8.5)
Urological	7 (6.6)
Started dialysis with catheter	61 (58)
Predialysis Hemoglobin; mean (95% CI)	8.8 (8.5 - 9.1)
Predialysis Albumin; mean (95% CI)	3.7 (3.6 - 3.8)
Body mass index; mean (95% CI)	25 (24 -26)

Autoimmune: glomerulonephritis, Ig A nephropathy, vasculitis, etc. Genetic: Alport Syndrome (n = 6) and Polycystic disease (n = 3)

The time between the referral to the surgeon and the access creation was from 10 to 20 days. The median time to the first puncture was 21.5 days, IR 14-31. Time of cannulation of failed and non-failed fistulas did not differ. As expected, patient age influenced the proportion of failures. Patients above 65 years had 46% of AVF failure, whereas younger patients had 24% of AVF failure (OR: 2.64, 95% CI: 0.98-7.12; *p* = 0.051).

Only 14 of the 113 AVFs (12.4%) showed primary failure. The other 99 AVFs were cannulated. From this group, 18 failed during the first year of use. Then, the FPP rate - which does not include PF - was 80.9% (SE=4.1).

As shown in Table 2, primary unassisted patency rate at 12 months (which included PF) was 70.6%, despite the high incidence of diabetics (39%) and lower AVFs (65%). In the logistic regression analysis, that also comprised PF AVFs, diabetes was a strong independent predictor of AVF failure in univariate analysis: OR=3.3, 95%CI 1.38-7.88, p = 0.007. The AVF forearm location (lower) adjusted by DBT also proved to be highly significant: OR=3.03, 95CI%; 1.05 - 8.76, *p* = 0.04. The patency rate difference between DBT and non-DBT was also highly significant. In non-DBT patients, the patency rates of lower and upper AVFs were similar. By contrast, the patency rate of DBT patients with lower AVFs was under 50%.

**Table 2:** Patency rates

	RATE (%)	Standard Error	*p* value
PRIMARY UNASSISTED			
OVERALL; n = 113	70.6	4.4	
Non-Diabetics; n = 72	78.8	5.0	
Diabetics; n = 41	58.1	7.5	0.02
Upper AVF; n = 39	78.9	6.6	
Lower AVF, non-diabetics	74.5	6.1	
Lower AVF, diabetics	45.0	11.1	0.003

First year patency rates of autologous arteriovenous fistula (AVF). ***Primary Unassisted Patency:*** (Primary failure included). Time from access creation or placement until any first intervention (endovascular or surgical) crucial to maintain or restore blood flow, or reaching the end of study period.

## Discussion

The results of this prospective, long-term study suggest that, in spite of working with a high-risk population, a low PF rate and a high FPP rate are possible to achieve in almost all hemodialysis patients.

It is generally accepted that the operating surgeon is determinant in AVF success^10^^-^12. However, there are reports that refuse the influence of the vascular surgeon's experience^13^^-^17. Studies that report the work of a single trained surgeon are scarce^18^^-^20 and there are few comparative studies. One retrospective study^21^, which compared two surgeons, found that demographic characteristics and preoperative vascular mapping were similar in the included patients^21^. However, fistulae placement occurred in 98% for surgeon I and 71% for surgeon II. The surgeon factor was significantly predictive of AVF creation, and the overall access survival rate at 12 months was 58% for surgeon I and 47% for surgeon II^21^. By comparing the rates of several surgeons, a multi-centric Dutch study found that PF rates vary widely among hospitals^22^. In 12 institutions, PF varied from 8 to 50%. Only one of them was below our 12.4% rate. This variation was also observed in the latest meta-analysis^6^, where AVF PF rates varied from 5 to 62%. Also in that report, only 7 out of 37 studies showed a PF lower than 12%. Therefore, these results support our work hypothesis that the skills of the vascular surgeon would play an important role in AVF early functionality. Moreover, the creation of a wrist AVF was always our first option. If this failed, the next step was to try the elbow fistula. Given the higher patency rate obtained with brachiocephalic AVFs, it is tempting to speculate that our PF rate could have been even lower.

A 2003 paper by the DOPPS concluded that staff training and experience may be important for success when it comes to cannulating and maintaining an AVF^23^. Based on these results, our institution clearly established all procedures related to AVF handling. As demonstrated in a 2005 study^24^_,_ a proactive and organized approach could successfully result in optimum vascular accesses to most patients. After AVF creation all patients were followed weekly by the senior nephrologist, who assessed the fistula progress - or early failure - based on physical examination. Only one of the two most skilled technicians cannulated a novel AVF during the first trimester. After that, another technician was assigned to exclusively puncture the same AVF. However, if a vascular access was considered "very difficult", the first person assigned continued to handle that AVF exclusively. We believe this individualized handling of each AVF could explain our high functional patency rate, which compares favorably with previous reports^6^^,^^22^^,^. When this study began, the optimal timing to first cannulate an AVF was still unresolved^25^. The KDOQUI recommended waiting 4-6 weeks^4^, but the DOPPS results did not concur with this opinion-based recommendation^26^. Our study cannot answer that question. We can only mention that, although 75% of the AVFs were cannulated from 0 to 31 days after their creation, only 18 out of 99 AVFs (18.2%) failed during the first year, suggesting that a long wait may not be necessary. Recent studies have also agreed to that and suggest that early cannulation is not associated with early failure^27^. Instead, wall shear stress and neointimal hyperplasia are believed to be key factors for early failure^28^^,^^29^. We suggest that multiple risk factors play a role during this critical period and emphasize the need for further investigation.

When planning vascular access, patients' preferences should also be considered. It has been shown that even long-term catheter-dependent patients would prefer autologous vascular access^24^. In the current study, all of our incident patients preferred the autologous AVF after thorough and adequate information. This study also showed a high primary unassisted patency after 12 months. Nevertheless, diabetic patients had a higher failure rate, especially if the vascular access was in the wrist, which agrees with previous work^30^. According to experts opinions^31^^,^^18^, the first option in DBT patients should be a forearm vascular access. This option decreases the patient's potential number of AVFs and increases the probability of steal syndrome. A thorough Doppler ultrasound (DUS) examination before surgery could lower that probability^32^. According to current guidelines, DUS should be carried out before and during the maturation time to assist the working team in making a decision on which vessels to utilize, monitoring maturation, and when and where should AVF be cannulated. Therefore, we acknowledge that the lack of Doppler exam and of a control group constitute the major limitations of our work. Furthermore, our definition of AVF maturation is limited to our clinical assessment. The major highlight of the current study is that a single surgeon operated with the same staff throughout the duration of the study.

In conclusion, our study suggests that a suitable, long-term permeable arteriovenous fistula is feasible in almost all hemodialysis patients. A skilled vascular surgeon and an experienced, committed hemodialysis staff play a relevant role in reaching this objective.
